# Follistatin-like 1 in development and human diseases

**DOI:** 10.1007/s00018-018-2805-0

**Published:** 2018-03-29

**Authors:** Andrea Mattiotti, Stuti Prakash, Phil Barnett, Maurice J. B. van den Hoff

**Affiliations:** Department of Medical Biology, Academic Medical Center, Meibergdreef 15, 1105 AZ Amsterdam, The Netherlands

**Keywords:** Cardiovascular disease, Cancer, Immune disease, Inflammation, Fibrosis, Obesity, Pulmonary disease, Signal transduction, Glycosylation, miRNA

## Abstract

Follistatin-like 1 (FSTL1) is a secreted glycoprotein displaying expression changes during development and disease, among which cardiovascular disease, cancer, and arthritis. The cardioprotective role of FSTL1 has been intensively studied over the last years, though its mechanism of action remains elusive. FSTL1 is involved in multiple signaling pathways and biological processes, including vascularization and regulation of the immune response, a feature that complicates its study. Binding to the DIP2A, TLR4 and BMP receptors have been shown, but other molecular partners probably exist. During cancer progression and rheumatoid arthritis, controversial data have been reported with respect to the proliferative, apoptotic, migratory, and inflammatory effects of FSTL1. This controversy might reside in the extensive post-transcriptional regulation of FSTL1. The FSTL1 primary transcript also encodes for a microRNA (miR-198) in primates and multiple microRNA-binding sites are present in the 3′UTR. The switch between expression of the FSTL1 protein and miR-198 is an important regulator of tumour metastasis and wound healing. The glycosylation state of FSTL1 is a determinant of biological activity, in cardiomyocytes the glycosylated form promoting proliferation and the non-glycosylated working anti-apoptotic. Moreover, the glycosylation state shows differences between species and tissues which might underlie the differences observed in in vitro studies. Finally, regulation at the level of protein secretion has been described.

**Electronic supplementary material:**

The online version of this article (10.1007/s00018-018-2805-0) contains supplementary material, which is available to authorized users.

## Introduction

Follistatin-like 1 (FSTL1) is a glycoprotein of the secreted protein acid and rich in cysteine (SPARC) family. In the literature, FSTL1 is referred to by many different names [1]. Two groups independently discovered FSTL1 and named it (I) transforming growth factor (TFG)-β-induced clone 36 (TSC36) isolated from mouse osteoblasts [2] and (II) Follistatin-related protein (FRP) secreted from rat glioma [3]. FSTL1 comprises a secretion signal, a Follistatin- and a Kazal-like domain, two EF-hand domains, and a von Willebrand factor type C domain (http://www.uniprot.org/uniprot/Q12841). Comparison of the human (Genbank: Q12841, 308 Aa) and mouse (Genbank Q62356; 306 Aa) protein sequences shows that the secretion signal (human: Aa 1–20 and mouse: Aa 1–18) is most species variable, whereas the remaining 272 Aa shows a very high degree of similarity (94.4%). In the sequence of mouse Fstl1, three potential sites for N-glycosylation and two for O-glycosylation are present and in vitro studies have shown that only the three aspartate residues Asp^142^, Asp^173^, and Asp^178^ are N-glycosylated. Moreover, glycosylation at these sites shows cell-type specificity [4]. N-glycoproteome analysis on human blood plasma identified only one glycosylated form of FSTL1 in which two (Asp^175^ and Asp^180^) of the three sites are used [5]. From gastrulation onward, Fstl1 mRNA is broadly expressed throughout the entire mouse embryo and its expression becomes restricted to the mesenchymal component of most tissues at the end of gestation [6]. In the adult mouse, the highest levels of Fstl1 mRNA are found in heart, lung, and subcutaneous white adipose tissue [7]. Interestingly, the expression of FSTL1 changes with respect to its level and pattern during various diseases, including cardiovascular disease [8–15], cancer progression [16–22], and systemic autoimmune diseases [23–26]. Analysis of the exomes of over 60,000 individuals revealed that the estimated probability of loss-of-function intolerance is 0.96 (http://exac.broadinstitute.org/gene/ENSG00000163430), reflected in the finding that homozygous loss-of-function mutations is never observed and heterozygous ones have only been described in 35 individuals. In line with this, functional disruption of Fstl1 in mice was found to result in respiratory distress and death within hours after birth. Gene knock-out mice display a phenotype that appears to suggest an important inhibitory role of FSTL1 in BMP signaling [27, 28]. Multiple TGFβ superfamily receptors as well as disco-interacting protein 2 homolog A (DIP2A) have been shown to interact with FSTL1 [28–30]. However, using other transgenic models, FSTL1 has been implicated in multiple signaling pathways and its role during diseases remains unclear [1]. Recently, a study on the role of FSTL1 in cardiac regeneration showed different effects on cardiomyocyte proliferation and protection from apoptosis depending on the post-translational modification of the protein [13], opening a new perspective on the interpretation of previously seemingly contradicting data. Moreover, post-transcriptional regulation plays an important role in the expression of the protein [31] and multiple functional miRNA-binding sites have been identified in the 3′UTR of FSTL1 mRNA [24, 32, 33]. For this reason, we present a review of the studies that have reported various roles of FSTL1 in development and disease while trying to clarify the sometimes contradictory results.

## Cardiovascular system

**Fig. 1:**
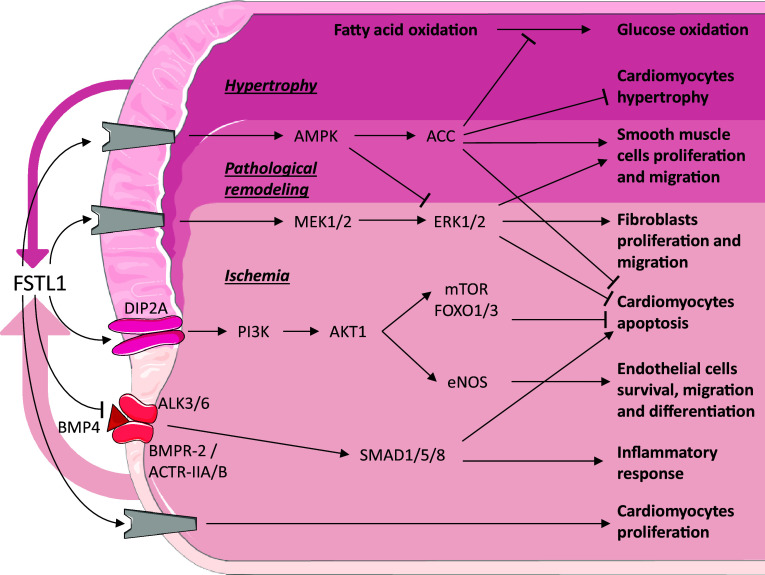
Follistatin-like 1 in cardiovascular disease. Schematic representation of the known signaling pathways interacting with FSTL1 in cardiovascular disease. Grey components indicate unknown receptors. The coloured arrows denote the secreted Fstl1 (width relates amount). Coloured area defines different conditions. Image adjusted from http://smart.servier.com

Cardiovascular disease (CVD), such as heart failure (HF) and coronary artery disease, is a group of disorders that affect the heart and blood vessels. CVD is a leading cause of death in Western countries [34]. For the sake of clarity, we have included a summary figure to accompany the text below (Fig. 1).

### Clinical relevance

In humans, circulating concentrations of FSTL1 increase during cardiac and vascular diseases, such as HF [9, 35], HF with preserved ejection fraction (HFpEF) [15], acute coronary syndrome (ACS) [36], and chronic obstructive pulmonary disease [37]. In patients with ACS, the increase in the serum level of FSTL1 correlates with mortality [36]. Moreover, the levels of FSTL1 in the circulating blood were found to correlate with the severity of chronic HF [35, 38]. Although FSTL1 has a negative prognostic value with respect to HF, it was found that patients with end-stage HF who received a combination of a left ventricle assist device (LVAD) and pharmacological therapy, who had high levels of FSTL1 at the time of LVAD implantation, showed recuperation and recovery of ejection fraction [9]. This finding suggests that FSTL1 could be a therapeutic target for drug development and serum concentration of FSTL1 can be used as a prognostic biomarker for CVD [35, 39].

### Cardiac development

RNA-Seq data (https://www.ncbi.nlm.nih.gov/gene/11167) show that FSTL1 mRNA is expressed in the adult human heart, but the expression pattern of the mRNA has not been established. However, immunohistochemical data showed that FSTL1 is present in vascular endothelial cells of vessels located within the myocardium, in smooth muscle cells of larger vessels and at a low, but significant levels in cardiomyocytes [9]. Furthermore, the pattern of expression of FSTL1 during human cardiac development remains to be established. In mouse and chicken, Fstl1 is expressed throughout the entire heart during early development and with expression subsequently becoming largely restricted to the non-myocardial component, with low levels being expressed in the cardiomyocytes [6, 40]. Homozygous deletion of Fstl1 in mice results in an overall enlargement of the heart of neonates [27]. Whether this enlargement is due to hyperplasia or hypertrophy of the cardiomyocytes is not yet known, and is currently being evaluated.

### Myocardial infarction

During a myocardial infarction (MI), blood flow to a portion of the heart muscle is interrupted causing local ischemia when prolonged results in cardiomyocyte death [41]. The dying cardiomyocytes trigger an inflammatory response which is followed by a reparative process that results in the formation of non-muscular scar tissue, thus reducing cardiac contractility and output [42].

After inducing an MI in mice by permanent occlusion of the left anterior descending artery (LAD), Fstl1 is transiently highly expressed in the heart, especially in the ischemic zone. Fstl1 reaches its highest level of mRNA expression 1 week after MI and normalizes in the remote zone of the heart at 1 month after MI, while low levels of expression persist in the infarct zone [10, 14]. Non-myocardial cells have been shown to be the major source of Fstl1 in the heart, with low levels also being detectable in the cardiomyocytes [13, 14]. Because Fstl1 is predominantly expressed in the ischemic zone, the serum levels seem to be a reflection of the infarct size. This might also underlie the observed correlation between elevated FSTL1 levels and mortality in ACS patients [36]. However, other organs that serve as a source of FSTL1 cannot be excluded. During the healing process after an MI, the epicardium plays an important role as a source of signaling molecules and provides cells to the infarct zone [10, 43]. Fstl1 is transiently expressed in the epicardium overlaying the infarct tissue and in the derived mesenchymal cells that populate the infarct scar [10, 13].

In both permanent and transient LAD ligation mouse models, it was found that locally produced and circulating Fstl1 protein, assessed by overexpression of Fstl1 in the liver using a recombinant adenovirus, have positive effects on survival and regeneration leading to a significant reduction of the infarct size [8, 13]. To study the beneficial role of FSTL1, *E. coli* produced human FSTL1 was intravenously administrated in a murine model either before inducing ischemia or after reperfusion; to validate a therapeutic effect in a larger animal, a porcine model was subjected to 45 min of ischemia and 24 h of reperfusion and intracoronary administration of FSTL1 during the first 10 min after ischemia. In both models, administration of FSTL1 reduced ischemic damage and improved cardiac performance after reperfusion [11]. Applying a collagen patch to the infarcted heart ameliorated recovery, which was even further improved when this patch was enriched with epicardium-derived medium or bacterially produced recombinant FSTL1 protein. The addition of bacterially produced FSTL1 significantly decreases the infarct area and as a consequence increases survival after MI [13]. In pig, a collagen patch loaded with FSTL1 applied to the ventricle 1 week after ischemia/reperfusion was also found to promote the regenerative response [13]. In cultured neonatal rat cardiomyocytes (NRCM), recombinant protein reduced hypoxia/reoxygenation-induced apoptosis both directly via (I) the MEK1/2 (mitogen-activated protein kinase kinase) and ERK1/2 (extracellular signal-regulated kinase) signaling pathway, (II) the DIP2A, PI3K (phosphoinositide 3-kinase) and AKT1 (RAC-alpha serine/threonine–protein kinase) pathway with activation of downstream effectors mTOR and FOXO1/3 [8, 44] and (III) AMPK (AMP-activated protein kinase) phosphorylation, and indirectly via (IV) inhibition of BMP4-induced apoptosis and (V) reduction of pro-inflammatory cytokines expression [11]. Interestingly, a difference in the effect between bacterially and eukaryotically produced FSTL1 was observed in the study of Wei and colleagues [13]. It should be noted that bacterially produced proteins are not glycosylated, while eukaryotically produced proteins are. The non-glycosylated FSTL1 increased NRCM proliferation, while the glycosylated protein, in accordance with previous reports [8, 11], protected NRCM from peroxidase-induced apoptosis [13]. In vitro experiments showed that glycosylated FSTL1 promotes fibroblast proliferation and migration via ERK1/2 phosphorylation [14]. Deletion of *Fstl1* from a part of the fibroblast population using the S100A4–Cre mouse line did not affect cardiac function compared to control littermates. Upon the induction of an MI, however, the number of mice dying due to cardiac rupture within the acute phase, i.e., first 7 day post-MI, doubled from a 25% in wild type to 50% in *Fstl1*-depleted animals. Analysis of cardiac function did not reveal any significant differences, though the number of myofibroblasts was decreased and the synthesis and maturation of the extracellular matrix proteins were reduced [14], indicating that the initial reparative response is abrogated.

### Cardiac hypertrophy

Cardiac hypertrophy is characterized by the abnormal thickening of the wall of the heart as a result of an increase in the volume of the individual cardiomyocytes [45].

Fstl1 expression is upregulated in mice after transverse aortic constriction (TAC), which results in pressure overload-induced hypertrophy [12] or by aldosterone infusion which causes hypertension-induced HFpEF [15]. In both these models, cardiomyocytes are the major source of Fstl1 [12, 15]. Treatment of adult rat ventricular cardiomyocytes with recombinant glycosylated human FSTL1 produced in Sf9 insect cells abrogates the increase in protein synthesis and Nppa (also known as: ANF or ANP) expression induced by aldosterone stimulation, indicating that FSTL1 prevents hypertrophy in cardiomyocytes. In HFpEF mice, increased levels of circulating Fstl1, mediated by overexpression in the liver via adenoviral delivery, significantly reduce cardiomyocyte hypertrophy and ameliorate cardiac functions [15]. Specific deletion of *Fstl1* from cardiomyocytes using the alpha myosin heavy chain (αMHC)–Cre mouse line did not show structural or functional differences compared to control littermates [12, 15]. However, when these mice were challenged by TAC, cardiac hypertrophy was enhanced and ventricular performance decreased [12]. On the other hand, when these mice were challenged by uninephrectomy in combination with 4 weeks of contiguous infusion of aldosterone, HFpEF ensued [15]. Interestingly, in the latter model, the role of Fstl1 in the development of HFpEF was independent of the changes in cardiac fibrosis but crucial in the development of cardiac hypertrophy [15]. In vitro experiments showed that the inhibitory effect of Fstl1 on cardiomyocyte hypertrophy is mediated by AMPK and acetyl-CoA carboxylase (ACC) phosphorylation [12].

During cardiac disease, the energy consumption of cardiomyocytes changes from fatty acid to glucose [46]. When heart failure is induced by tachy-pacing in dogs, this metabolic switch is also observed. Treatment of these dogs with a single dose or long-term infusion (14 days) with glycosylated human FSTL1 (CHO cells) inhibited the pathological switch from fatty acid to glucose oxidation. This effect was transient, because after clearing FSTL1 from the blood, consumption of glucose increased and fatty acid consumption decreased. Moreover, the infusion of AMPK inhibitor neutralized the effect of FSTL1 [47].

### Vascular system

In some of the previously mentioned studies on MI and cardiac hypertrophy, mice also displayed a pro-angiogenic effect of Fstl1, though the underlying mechanism was not investigated [12–14]. A similar pro-angiogenic effect was also found in a mouse model of ischemic hind limb, in which Fstl1 expression was found to be induced in skeletal muscle cells [48]. Adenoviral-mediated overexpression of Fstl1 in the latter mouse model was found to stimulate revascularization. Interestingly, adenoviral-mediated overexpression of Fstl1 in non-ischemic muscle did not alter its vascularization. In this model of hind limb ischemia, glycosylated Fstl1 was found to promote endothelial cell survival, migration, and differentiation into a vascular network-like structure via phosphatidylinositol-3 kinase (PI3K), AKT, and endothelial nitric-oxide synthase (eNOS) activation [48]. At that time, the receptor conveying the extracellular Fstl1 signal into the cell was unknown, but recently, DIP2A has been identified as a potential cell-surface receptor upstream of PI3K and AKT1 phosphorylation [44]. Interestingly, an opposite effect of FSTL1 has been observed in smooth muscle cells (SMCs) during pulmonary hypertension [37] and vascular injury [49, 50]. In the latter two models, FSTL1 prevents pathological vascular remodelling, as a result of a decrease in SMC proliferation and migration which is mediated via the induction of AMPK and inhibition of ERK phosphorylation [37, 49, 50]. Studies on HUVEC cells showed that FSTL1 affects vascular endothelial cell polarization, but not migration and tube formation [51].

### Mitral valve disease

Mitral valve disease is a major cause of morbidity, heart failure, and death worldwide [52]. Deletion of *Fstl1* from the endothelial/endocardial lineage using the Tie2–Cre mouse line resulted in dysfunctional mitral valves, HFpEF and death [53]. The dysfunctional mitral valve leaflets became long and thick, suggestive of enhanced proliferation of the valve cells and formation of mesenchymal cells as a result of prolonged endocardial to mesenchymal transition, valve enlargement itself probably being due to enhanced TGF-β and BMP signaling. Whether the observed development of HFpEF is a direct consequence of deletion of *Fstl1* or a secondary effect of the dysfunctional mitral valve or the enhanced TGF-β and BMP signaling, is not yet clear [53]. It should be noted at this point that FSTL1 was not found in a meta-analysis of genome-wide association studies identifying 23 loci with suggestive evidence of association with mitral valve prolapse in the human population [54].

### In conclusion

FSTL1 is a secreted glycoprotein with both protective and regenerative capacities of which the circulating concentration increases during CVD. Overexpression or lowered expression (hypomorphic or heterozygous KO) of Fstl1 is tolerated in healthy animal models, whereas during pathological conditions, additional Fstl1 prevents extensive cardiac damage and abnormal vascular remodelling. A lack of Fstl1 exacerbates cardiac injury. Most importantly, Fstl1 affects multiple pathways in a cell-type specific manner and different effects are observed depending on the glycosylation state of the protein. These results suggest that FSTL1 may not just represent a biomarker, but could also be an interesting candidate for the development of new therapies in CVD.

## Cancer and tumours

**Fig. 2:**
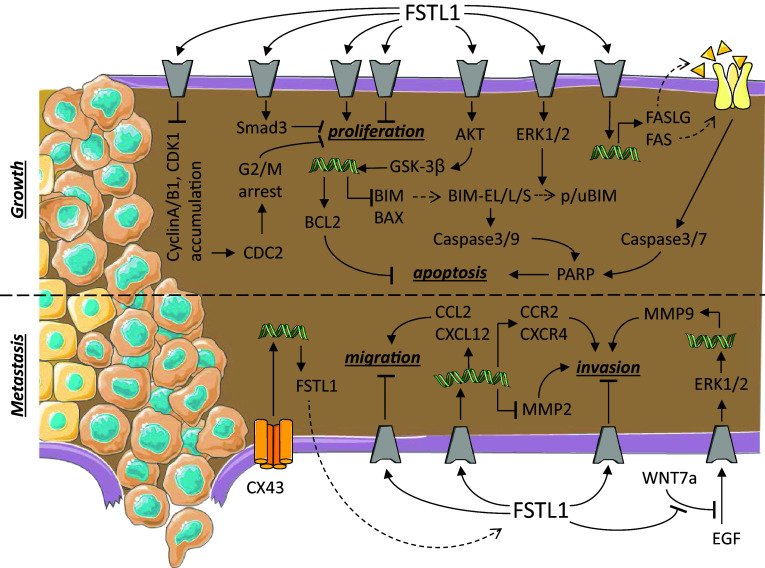
Follistatin-like 1 in cancer and tumour. Schematic representation of the known signaling pathways interacting with FSTL1 during cancer growth and metastasis. Grey components indicate unknown receptors. Helical structures represent gene expression. Dashed arrow indicates translation from mRNA to protein. The dashed line separates different processes: tumour growth and metastasis. Image adjusted from http://smart.servier.com

Cancer is characterized by an imbalance in growth regulation caused by genetic changes. When cancer cells do not migrate into the surrounding tissues, the tumour is referred to as a benign. Cancer cells become malignant when they acquire the additional property of migration, allowing them to invade other tissues, this being referred to as metastasis. When left untreated, tumour metastasis is the leading cause of death in cancer patients [1, 55, 56]. For the sake of clarity, we have included a summary figure to accompany the text below (Fig. 2).

### Clinical relevance

Compared to healthy tissue, the expression level of FSTL1 was found to be reduced in biopsies of various types of cancers, such as prostate [16], ovarian, endometrial [19], kidney [20, 21, 57], nasopharyngeal carcinoma (NPC) [22], and lung adenocarcinoma [58]. In endometrial and ovarian tumours, a trend between poorer prognostic characteristics and decreasing levels of FSTL1 was observed [19]. In clear-cell renal cell carcinoma (ccRCC), high levels of FSTL1 expression correlate with a favorable post-operative survival (hazard ratio = 0.325; *p* = 0.030). Knock-down of FSTL1 using retrovirus mediated short hairpin RNA in ccRCC cell lines resulted in anchorage independent growth and invasion. Further analysis showed that the tumour suppressor function of FSTL1 is mediated through repression of the NF-κB and HIF-2α signaling pathways [57]. Moreover, in line with these novel findings, a polymorphism in the second intron of the *FSTL1* gene, associated with downregulation of its expression, correlates with an increased risk of renal cell carcinoma and poor prognosis [21]. Interestingly, in nasopharyngeal carcinoma (NPC) cell lines and tumour biopsies, decreased levels of FSTL1 mRNA correlate with hypermethylation of CpG islands in its proximal promoter (− 166 to + 332 bp) and also with increased tumourigenicity. Restoring of FSTL1 mRNA levels using demethylating agents decreased tumourigenicity. The reduced levels of expression of Fstl1 in the NPC tumours are reflected in decreased FSTL1 serum levels [22]. A low level of expression of FSTL1 in lung adenocarcinoma patients has a poor prognostic value (hazard ratio = 2.09; *p* = 0.022), but no correlation was observed between FSTL1 expression and survival in squamous cell carcinoma [58].

On the other hand, increased expression of FSTL1 compared to healthy control tissue was observed in brain cancer cells [18], in fibroblasts but not in epithelial cells in colon tumours [59], in castration-recurrent prostate cancer [60], in most cases of hepatocellular carcinomas (HCC) [61], in head and neck squamous cell carcinoma (HNSCC) [62], and esophageal squamous cell carcinoma (ESCC) [63]. FSTL1 is higher expressed in metastatic brain tumours compared to primary breast cancer [64]. FSTL1 is not expressed at a detectable level in normal brain tissue and in diffuse-infiltrating astrocytes of grade II or III gliomas, but is expressed at high levels in grade IV gliomas, referred to as glioblastoma [18]. The level of FSTL1 was found to correlate with the malignancy of the astrocytoma and its coexpression with p53 has a negative prognostic value [18]. In HCC, high levels of FSTL1 correlate with larger tumours, advanced tumour/node/metastasis (TNM) stages, metastasis, and poor post-surgical outcomes (hazard ratio = 1.84; *p* = 0.015) [61]. The high levels of Fstl1 correlate with lower survival perspective in HNSCC and ESCC patients [62, 63].

Most cell lines derived from human tumours express lower levels of FSTL1 than immortalized non-tumourigenic fibroblasts [65]. In vitro transfection of fibroblasts (mouse NIH3T3 and rat 208F) using oncogenes like ras, myc, or fos induces a tumourigenic phenotype and downregulation of Fstl1 expression, suggesting a tumour suppressor role [2, 65, 66]. Comparison of human cancer cell lines with a difference in aggressiveness of their phenotype showed opposite outcomes: the prostate cancer cell line LNCaP shows lower FSTL1 expression levels compared to the more aggressive C4-2 variant [17], while less aggressive non-small cell lung carcinoma (NSCLC) cells express on average higher levels of FSTL1 compared to more malignant small cell lung cancer (SCLC) cells [67]. However, high variability in FSTL1 expression levels is observed in NSCL cell lines from both adeno- or squamous carcinomas.

Taken together, the data on the expression levels of FSTL1 in relation with its effect in different tumours are highly variable from respect to growth inhibition or induction and from invasiveness to immobility. As a consequence, the expression level of FSTL1 should be regarded with caution, and not be associated with a specific phenotype.

### Tumour growth

With respect to imbalanced tumour growth, contradictory results have also been reported upon in vitro manipulation of the level of expression of FSTL1 in cell lines.

Transfection of lung NSCLC cell lines (PC-14 and H446) [58, 67, 68], ovarian, endometrial [19], NPC [22], and breast cancer [64] cell lines with FSTL1 reduces their growth rate via an unknown signaling pathway. Overexpression of FSTL1 in ovarian or endometrial cells was also found to increase the rate of apoptosis via a death receptor-initiated pathway. In these cells, an increase in the mRNA of Fas cell-surface death receptor (FAS or First Apoptotic Signal receptor) and its ligand (FASLG) was found to result in an increase in cleaved (activated) PARP, Caspase-3, and Caspase-7 [19]. Downregulation of FSTL1 in ccRCC [57] and breast cancer [64] cells promotes proliferation. Phosphorylation of Smad2/3 is one of the pathways that mediate FSTL1 inhibition of proliferation [64]. Injection of NPC cells stably transfected with FSTL1 in nude mice showed a similar tumour incidence compared to non-transfected cells. However, the tumour growth rate was lower in FSTL1-transfected NPC cells compared to control cells [22]. Similar in vivo results were observed in NSCLC; tumour growth was inhibited when FSTL1 was overexpressed in the CL1-5 cell line and promoted when FSTL1 expression was downregulated in the CL1-0 cell line [58].

Decreasing the level of FSTL1 in human NSCLC cell lines (NCI-H460 and A549) using an siRNA induced the accumulation of cell cycle proteins, such as cyclin A, cyclin B1, CDK1, and phosphorylated CDC2 and caused G2/M arrest [69]. In this same study, it was also reported that apoptosis increased. The increase in apoptosis was mediated by a decrease in ERK1/2 phosphorylation, an increase in both cleaved (activated) Caspase-3, Caspase-9, and PARP, and an accumulation of the pro-apoptotic factor BIM-EL [69]. Overexpression of FSTL1 in a human hepatocellular carcinoma (Huh7) cell line promoted its expansion, being the result of increased proliferation and inhibited apoptosis. The inhibition of apoptosis was found to be the result of activated AKT/GSK-3β signaling, with as consequence an increase in the anti-apoptotic protein BCL-2 and a decrease in the pro-apoptotic BIM and BAX proteins [61]. Similar results were reported in ESCC cells (KYSE-150), where inhibitory effect of FSTL1 on the BMP-signaling pathway and chemoresistance could be demonstrated [63]. Overexpression of FSTL1 in mouse MC3T3 osteoblast precursor cells [65] or rat 208F fibroblasts [66], on the other hand, did not affect cell morphology or proliferation. Although downregulation of FSTL1 in a squamous cell carcinoma (SCC12) cell line did not affect the proliferation rate in vitro, they were found to form larger tumours when injected in nude mice [62].

### Metastasis

Overexpression of FSTL1 in lung (PC-14 line and H446) [67, 68], ovarian, endometrial [19], or NPC [22] cell lines not only decreased their proliferative capacity, but also their ability to migrate and invade flanking tissues. This reduction in migratory capacity was accompanied by a reduction in metalloproteinase-2 (MMP-2) expression [19, 66]. In line with these results, downregulation of FSTL1 in ccRCC cells was shown to lead to a reduced migratory capacity and tissue invasion [57].

**Fig. 3:**
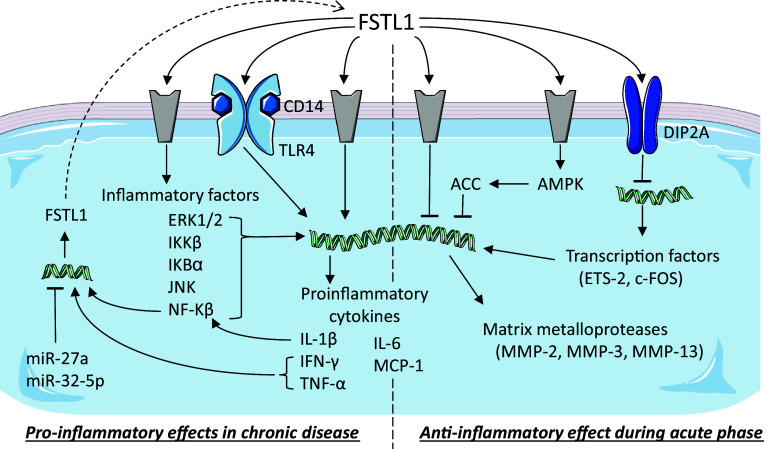
Follistatin-like 1 in immune diseases. Schematic representation of the known signaling pathways interacting with FSTL1 during inflammatory processes. Grey components indicate unknown receptors. Helical structures represent gene expression. Dashed arrow indicates translation from mRNA to protein. The dashed line separates the two opposite effects of FSTL1: pro- and anti-inflammatory. Image adjusted from http://smart.servier.com

Downregulation of FSTL1 in human melanoma cells using a siRNA results in the inhibition of the expression of genes associated with migration, such as CCL2 and CXCL12, and with the formation of bone metastasis, such as CCR2 and CXCR4 [70]. In line with these observations, stimulation of these melanoma cells with recombinant glycosylated FSTL1 induced their migratory capacity and differentiation into a bone phenotype. FSTL1 is also lower expressed in the breast cancer cell line MDA-MB-231 compared to its metastatic version 231-BR [64]. In vivo studies, further showed that injection of an FSTL1 siRNA into a subcutaneous tumour suppressed tumour growth and the formation of bone metastasis, increasing mouse survival compared to injections with a control siRNA [70]. Overexpression of FSTL1 in ESCC cells (KYSE-150) promoted tumour growth and metastasis in vivo. In vitro analysis showed that inflammation (Fig. 3) and epithelial-to-mesenchymal transition processes were strongly affected by FSTL1 [57, 63]. A recent study on the metastatic effect of FSTL1 showed that FSTL1 specifically interacted with WNT7a and antagonized its inhibitory effect on endothelial growth factor-mediated ERK phosphorylation which in turn induced the expression of MMP9 [62], a prerequisite for cell migration. Overexpression of Connexin-43 in pulmonary giant carcinoma cell inhibited their metastatic capacity. This inhibitory effect could be reversed by adding antibodies against FSTL1 protein [71]. These observations need to be regarded with caution, as they might be pointing to two independent signaling pathways regulating cell invasion.

### In conclusion

Due to the heterogeneity in the different cell lines and cancers and the complexity of the multiple mechanisms underlying tumour development, our knowledge on the role of FSTL1 during cancer development and progression is still fragmented and limited. The origin of the tumourigenic cell line appears to be a main determinant for the different, sometimes opposite, effect of FSTL1.

## Immune diseases

The immune system is the collective of tissues, cells, and processes that provide a specific response to protect the organism upon invasion by organisms, foreign cells, and toxins. Dysfunction of components of the immune system often results in immune disorders. Systemic autoimmune diseases (SADs) are disorders characterized by loss of function or destruction of normal tissues due to autoimmunity which is specific to each disease. The role of FSTL1 during inflammatory processes has been studied in several models and reported to be both pro- and anti-inflammatory. For the sake of clarity, we have included a summary figure to accompany the text below (Fig. 3).

### Clinical relevance

High serum concentrations of FSTL1 are found in many SADs, such as rheumatoid arthritis (RA) [23, 24], in particular juvenile RA with systemic onset [25], osteoarthritis (OA) [26], and Sjögren’s syndrome [23]. Synovial tissue of RA patients expresses FSTL1 at high levels, which correlate positively with clinical and serological parameters of disease [72]. In serum of RA patients, antibodies directed against FSTL1 appear more frequently (30%) than in other SADs, such as systemic sclerosis (17%), systemic lupus erythematosus (10%), and Sjögren’s syndrome (10%). Antibodies against FSTL1 were not found in patients with OA or polymyositis/dermatomyositis or healthy controls [73]. Besides the elevated serum levels of FSTL1 in RA, no association of polymorphisms in Fstl1 with susceptibility to RA has been found to date [74], suggesting that overexpression of FSTL1 is a consequence of RA rather than a cause.

Patients with Kawasaki disease present with inflammation of blood vessels throughout their body. Plasma levels of FSTL1 are significantly elevated in acute Kawasaki disease patients compared to control subjects. Upon intravenous immunoglobulin therapy, levels of FSTL1 were found to slowly decrease over time [75]. Interestingly, FSTL1 levels correlate with a high risk of developing coronary artery aneurysm [75], which is a major cause of morbidity and mortality [76].

Lumbar disc herniation (LDH) is a medical condition affecting the spine in which a rupture in the outer fibrous ring of an intervertebral disc allows the soft central portion to bulge out beyond the damaged outer rings causing inflammation. Serum levels of FSTL1 are higher in LDH patients compared to scoliosis patients and healthy controls and correlate with the amount of pain on a visual analogue scale [77].

### Arthritis

To evaluate the role of FSTL1 in arthritis, two different animal models are used that result in apparently contradictory effects. In the collagen-induced arthritis (CIA) model, the mice are immunized by intradermal administration of an emulsion of complete Freund’s adjuvant and bovine type II collagen, and 21 days later, a booster or secondary immunization is given [78]; in this mouse model, FSTL1 was found to promote inflammation. In the anti-type II collagen antibody-induced arthritis (CAIA) model, the mice are intraperitoneally injected with a cocktail of monoclonal antibodies directed against type II collagen, and 72 h later, they are injected with endotoxin (LPS) [79]; in this acute, destructive arthritis model FSTL1 was found to be anti-inflammatory. The different effects reported in these two models of arthritis are likely due to the differences in disease development and in the evaluated therapeutic potential of FSTL1; in the CIA model, adenovirus mediated overexpression of mouse Fstl1 (Ad-mFstl1) and in the CAIA model administration of recombinant non-glycosylated FSTL1.

Fstl1 is normally expressed in synovium, but in early stage of CIA, Fstl1 becomes highly overexpressed in fibroblasts at the margin of the eroding bone and in cells of the mesenchymal lineage including osteocytes, chondrocytes, and adipocytes but not in cells of the hematopoietic lineage, like macrophages, neutrophils, or T cells [25, 80, 81]. Ad-mFstl1 increased the secretion of pro-inflammatory cytokines, IFN-γ, tumour necrosis factor alpha (TNF-α), interleukin-1β (IL-1β), and 6 (IL-6), resulting in the induction of synovitis (inflammation of the synovial membrane) with infiltration of inflammatory cells in the synovium and surrounding tissue. Both in vitro and in vivo experiments showed that IL-1β induces Fstl1 expression through NF-κB [82], suggesting a positive feedback between the inflammatory response and Fstl1 expression. In CIA mice, Ad-mFstl1 exacerbates severity of arthritis, while administration of anti-mFstl1 IgG neutralizes endogenous Fstl1 and reduces the severity of disease [81, 82]. A similar pro-inflammatory effect, mediated via the CD14-TLR4 pathway, was also found in CIA mice in which Fstl1 was delivered by genetically modified-T cells [83]. Moreover, it was observed that FSTL1 functioned in a species-specific manner: recombinant human but not mouse protein, both produced in human cells (HEK293 and HT-1080), induces IL-6 expression in cultured synovial cells from RA patients but not in mouse NIH-3T3 cells and vice versa [83]. Interestingly, the mouse and human proteins are very similar (94.4%), and although the effect difference may lie in this small difference, perhaps, a differing glycosylation state of the two proteins plays a major role in determining their observed biological activities.

In the CAIA mouse model, an increase in Fstl1 expression is observed during early stages of arthritis. In this model, the administration of *E. coli*-derived recombinant human FSTL1 showed anti-destructive and anti-inflammatory effects, reducing synovial cellular infiltration and retaining cartilage proteoglycan. Treatment of synovial cells and joints with FSTL1 resulted in downregulation of the transcription factors c-Fos and Ets-2 and downstream matrix metalloproteases, such as Mmp-3 [84, 85]. In line with these observations, upregulation of these genes was observed when cells were treated with antibodies directed against FSTL1 that were obtained from mouse or from RA patients [84, 85]. Interestingly, both c-FOS and ETS-2 are expressed at higher levels in RA patients than in healthy controls [86]. In vitro experiments showed that downregulation of c-FOS is mediated by FSTL1 binding to DIP2A receptor [84]. Curiously, during differentiation of bone-marrow-derived macrophages into osteoclasts, Fstl1 induces the transcription of c-Fos [87], suggesting a different role of Fstl1 during normal development and in pathological conditions. In this CAIA model, like in the CIA model, the expression of IL-6 was reduced [85], but it is important to note that in the CAIA mouse model, IL-6 is not responsible for the progression of arthritis [88].

### Pro-inflammatory effect

In a mouse model for bacterially induced septic shock, endotoxin (LPS) administration in the rear footpads induces the expression of both Fstl1 and IL-1β. In control mice and in transgenic mice, where *Fstl1* was removed by tamoxifen administration, IL-1β remained low or undetectable [89]. Overexpression of FSTL1 in cultured monocytes and macrophages or in the septic shock mouse model induces expression of caspase-1 and NLRP3 (nucleotide-binding domain leucine-rich repeat containing (NLR) family, pyrin domain containing 3), confirming that FSTL1 mediates pro-inflammatory events [89]. In cultured adipocytes, macrophages, and nucleus pulposus cells, the pro-inflammatory effect of FSTL1 is mediated via the signaling cascade IKKβ, IκBα, NF-κB, JNK, and ERK1/2 resulting in the induction of expression of IL-1β, IL-6, TNF-α, MCP (monocyte chemotactic protein)-1, COX (cyclooxygenase)-2, MMP-13, and iNOS (inducible nitric-oxide synthase) [22, 77, 87, 90]. Moreover, stimulation of nucleus pulposus cells with TNF-α induces FSTL1 secretion [77], again suggesting a positive feedback loop on the inflammatory response. Treatment of bone-marrow-derived macrophages with glycosylated recombinant FSTL1 increases cell proliferation in a dose-dependent manner [87]. Together. these data suggest that FSTL1 promotes inflammation by inducing not only cytokine production, but also proliferation of inflammatory cells.

### Anti-inflammatory effect

Observing lowered expression levels of Fstl1 using a hypomorphic mouse model revealed no effect on the levels of pro-inflammatory cytokines, such as TNF-α, IL-6, and IL-1β, in kidney [91]. However, in renal injury mouse models, circulating Fstl1 appeared to regulate the immune response: in a cisplatin nephrotoxicity model Fstl1 inhibits the synthesis of pro-inflammatory cytokines, like IL-1β [91], and in a subtotal nephrectomy model Fstl1 decreases TNF-α, IL-6, and MCP-1 (monocyte chemotactic protein-1) expression [92]. In vitro experiments on human, mesangial cells showed that this anti-inflammatory effect is mediated by phosphorylation of AMPK and activation of acetyl-CoA carboxylase (ACC) [92].

Upon cardiovascular injury, administration of recombinant glycosylated human FSTL1 produced in Sf9 insect cells reduced pro-inflammatory cytokine expression. In vitro studies on NRCM and macrophages showed that this inflammatory response is mediated both (I) by inducing AMPK phosphorylation and downstream ACC activation and (II) by inhibiting BMP4 signaling which otherwise increases TNF-α and IL-6 expression via pSmad1/5/8 [11]. After ligation of the femoral artery in mice, in which Fstl1 expression from the muscle cells was deleted using the muscle creatine kinase (MCK)–Cre mouse line, the inflammatory response was enhanced. This was evidenced by an increase in the expression of the pro-inflammatory cytokines TNF-α, IL-1β, and MCP-1 and in the infiltration of monocytes and macrophages [50].

In host-versus-graft disease, FSTL1 was also found to play a role. An upstream morphogen of FSTL1, TGF-β, is known to play a central role in allograft tolerance [2, 93]. In line with this, FSTL1 has been shown to be induced in donor specific blood transfusions after heart transplantation probably as a result of infiltrating CD8^+^ T cells. It is of relevance to note that the infiltration of T cells was not related to an inflammatory response due to surgery. Intravenous administration of an adenovirus expressing FSTL1 leads to a reduction in expression of pro-inflammatory cytokines (IL-17A, IL-6, and IFN-γ) and prolonging survival of transplanted patients [93]. A similar effect is also observed during cancer progression, in which FSTL1 plays an important role in immune dysfunction regulating thymocyte maturation: in vivo inhibition of Fstl1 increases the tumour-specific CD8^+^ T-cell response [70].

### In conclusion

Taken together FSTL1 was observed to have a dual function during inflammatory processes, acting as an anti-inflammatory factor in the acute phase but having a pro-inflammatory effect in the long term and in chronic diseases. This is likely due to activation of different signaling pathways: initially, FSTL1 binds the DIP2A receptor and prevents tissue degradation by MMPs through the downregulation of the transcription factors c-FOS and ETS-2; subsequently, FSTL1 activates the inflammatory response via the TLR4/CD14 pathway, the activation of the AMPK pathway, and the inhibition of BMP-signaling pathway. However, it cannot be excluded that additional endogenous or exogenous factors are involved in the regulation.

## FSTL1 in other diseases

### Fibrosis

Fibrosis refers to the formation of excessive connective tissue in an organ or tissue during a reactive and/or reparative process. Compared to healthy tissue, FSTL1 expression increases in patients with idiopathic pulmonary fibrosis and in mouse models such as bleomycin-induced lung injury [94–96], CCl_4_-induced liver injury and in kidney after unilateral ureteral obstruction [97]. Haplodeficiency of *Fstl1* or reduced expression of Fstl1 using siRNA results in a reduction in collagen accumulation in both lung and liver injury [95, 97]. The effect on fibrosis is most probably due to disruption of the TGF-β/BMP balance by FSTL1 because of the ability of FSTL1 to inhibit Smad1/5/8-mediated BMP4 signaling and of the stimulation of expression of FSTL1 via Smad2/3 mediated TGF-β1 signaling [28, 95]. During nephrectomy, overexpression of circulating Fstl1 by adenovirus mediated delivery reduces kidney fibrosis formation and the expression of fibrosis markers, such as TGF-β1, collagen-I, collagen-III, and connective tissue growth factor [92]. It is important to note that unlike initial studies, in which the mice where analyzed 1–2 weeks after induction of fibrosis [95, 97], later studies analyzed mice 2 months after surgery [92]. This opposite effect on fibrosis development is in line with studies in immune diseases, in which FSTL1 also shows an opposite effect during the acute or chronic phases of disease.

### Lung development and asthma

During mouse development and in the adult mouse, Fstl1 mRNA is expressed in mesenchymal cells of the lung, vascular and airway smooth muscle cells, goblet cells of the airway epithelium and in the endothelial cells [6, 98]. Upon functional disruption of Fstl1, neonates die at birth due to respiratory distress, displaying tracheomalacia, hypoplastic, and absence of tracheal cartilage rings. Within the lung tissue, a thickening of the alveolar walls and a reduction in airspace were found. Moreover, differentiation of the airway epithelium seems to be impaired as seen in the reduced level in mature surfactant protein [27, 28].

Asthma, a chronic respiratory disease, is characterized by airway inflammation, remodelling, and hyper-responsiveness. Proteomic analysis of sputum of patients with asthma revealed that FSTL1 is one of the highest expressed proteins [99]. Histological analysis of post-mortem human lungs of asthma patients showed expression of FSTL1 in alveolar macrophages [100]. Moreover, the elevated levels of FSTL1 were found in blood plasma and bronchoalveolar lavage fluid in asthma patients compared to healthy controls [101]. The FSTL1 concentration negatively correlates with lung function parameters and positively with airway remodelling markers. Interestingly, plasma levels of FSTL1 significantly decrease and return to control levels 1 month after treatment with inhaled corticosteroids and long-acting β-agonist therapy and/or oral leukotriene receptor antagonist therapy [101]. Along the same line, mice chronically, but not acutely, challenged with an allergen showed an increase in Fstl1 expression [100]. Haplodeficiency of *Fstl1* [102] or deletion of *Fstl1* from macrophages/myeloid cells using the Lys–Cre mouse line [100] reduced inflammation (Fig. 3) and airway remodelling in OVA-challenged mice, while opposite effects are observed when mice were treated with recombinant glycosylated FSTL1, though in this study, it is not clear whether human or mouse protein was used [100]. Increased expression of Fstl1 in a chronic but not in an acute mouse model of asthma is in line with previously reported results suggesting different roles of FSTL1 during acute and chronic inflammation in arthritis [100].

Autophagy and epithelial-to-mesenchymal transition are two associated biological processes [103]. Autophagosome formation was found to be increased in human bronchial epithelial cell line 16HBE stimulated with recombinant FSTL1 protein using electron microscopical analysis and autophagy biomarkers (Beclin-1 and microtubule-associated protein 1A/1B-light chain 3). FSTL1 stimulation of these cells induced the switch from E-cadherin to N-cadherin expression, pointing endothelial-to-mesenchymal transition. Interestingly, when these cells were also treated with an autophagy inhibitor, not only autophagy but also endothelial-to-mesenchymal transition was attenuated [102].

### Obesity

Serum levels of FSTL1 were found to correlate with the body mass index with FSTL1 higher in overweight and obese subjects than in controls [90]. During differentiation of pre-adipocytes (3T3–L1) into adipocytes, Fstl1 showed a transient short high level expression to become subsequently downregulated to background levels at both the mRNA and protein level [7, 104]. Either by preventing this initial peak in Fstl1 expression or by maintaining high levels of Fstl1 in the culture medium during induced adipogenesis, the differentiation of 3T3–L1 cells was blocked [104]. Two different mechanisms have been identified linked directly to the regulation of Fstl1 expression. Recently, it was discovered that the secretion of Fstl1 was regulated via cilia. When genes essential for ciliogenesis, BBS4 or IFT88, were knocked-down in 3T3–L1 pre-adipocytes, the levels of Fstl1 mRNA and protein were downregulated and the cells failed to undergo correct adipogenesis [104].

The second mechanism involved the downregulation of the pro-inflammatory cytokines, IL-6, IL-8, and MCP-1, during the differentiation of 3T3–L1 cells into adipocytes [90]. The addition of TNFα to 3T3–L1 adipocytes induced their de-differentiation and was accompanied by a re-expression of Fstl1 mRNA and protein [7] and an upregulation of the pro-inflammatory cytokines [90]. The changes in the ratio of pro- and anti-inflammatory cytokines are thought to underlie the chronic inflammation observed in obesity, which leads to insulin resistance and other obesity-associated diseases [105]. This closely resembles the affects reported in arthritis, pointing to a role of FSTL1 in the regulation of the balance of pro- and anti-inflammatory cytokines.

Another, as yet, unexplored alternative mechanism could be via micro RNA (miRNA) regulation. Like in the differentiation of adipocytes, a similar downregulation of Fstl1 is observed during in vitro myogenesis of C2C12 myoblasts. In myoblasts, the downregulation of Fstl1 mRNA is regulated by muscle-specific miR-206 [32]. Furthermore, during adipogenesis, regulation of gene expression levels by microRNAs has been reported [106], and candidate miRNA-binding sites can be found when the 3′UTR of FSTLl1 is scanned for potential binding sites of microRNAs (Supplemental Table 1).

### Central nervous system

During mouse development, Fstl1 is locally expressed in all the component of the central nervous system [107] and it is involved in the radial glial scaffold formation [108]. In the dorsal root ganglia, Fstl1 is involved in maintenance of the normal threshold of somatic sensation: it is secreted from afferent axons and it activates the α1 subunit of the Na^+^,K^+^-ATPase, suppressing synaptic transmission. Neural deletion of Fstl1 using Na_v_1.8–Cre mice causes hypersensitivity of both wide dynamic range neurons and nociceptive neurons [109].

### FSTL1 and microRNAs

MicroRNAs (miRNAs) are short (~ 22 nt) endogenous noncoding RNAs that regulate messenger RNA (mRNA) degradation and/or translational repression [110]. Several studies have highlighted the relevance of the miRNA and miRNA-binding sites during human disease [111]. miR-198 is encoded in the 3′UTR of human FSTL1 primary transcript [112]. The FSTL1 mRNA, therefore, not only encodes the FSTL1 protein, but also an miRNA. Though the FSTL1 protein has been highly conserved during evolution from tick to human [29], the encoded miRNA is only found in primates. Moreover, in silico analysis revealed multiple miRNA-binding sites in the 3′UTR of the FSTL1 mRNA of which three have been functionally analyzed (miR-206 [32], miR-32-5p [33], and miR-27a [24]) (Table 1). A list of predicted miRNA-binding sites in the 3′UTR of human FSTL1 with associated clinical relevance is provided in Supplemental Table 1.

**Table 1:** MicroRNA-binding sites in FSTL1 gene

Human microRNA	Binding position in 3′UTR	Biological relevance
miR-27a	1537	Inflammation [24]
miR-32-5p	142	Inflammation [33]
miR-206	2101; 2375	Myogenesis [32]

List, position, and biological processes of the verified microRNA-binding sites in the 3′UTR of FSTL1 gene

In the normal healthy human epidermis, FSTL1 mRNA is expressed, but the protein is present at low to almost undetectable, levels. Contrary to this, miR-198 is expressed in this tissue. During wound healing, a switch from miR-198 to FSTL1 protein expression is observed without a change in the FSTL1 mRNA level. TGF-β1 indirectly regulates this switch via KH-type splicing regulatory protein (KHSRP). In the healing skin, FSTL1 protein promotes keratinocyte migration during re-epithelization. Interestingly, in chronic non-healing ulcer wounds in patients with diabetes mellitus, no FSTL1 protein expression is observed [113]. Disregulation of the ratio FSTL1 versus miR-198 is observed in head and neck squamous cell carcinoma, in which FSTL1 protein persists with the afore-mentioned consequences [62]. MiR-198 is also involved in suppressing colorectal cancer growth [114] and lung adenocarcinoma A549 cell proliferation [115], but the relation between FSTL1 and miR-198 in this cancer type has not yet been studied.

MiR-206 is one of the most abundant miRNAs expressed during skeletal myogenesis [116]. In chicken, the expression of FSTL1 (also referred to as Flik) is downregulated in the embryo during myotome formation suggesting a role during myogenesis [117]; however, the role of FSTL1 during this process has not been investigated. The group of Tapscott showed that skeletal muscle cell differentiation is coordinated by the transcription factor MyoD, and expression of FSTL1 is reduced [118]. This process appears to be mediated by the induction of miR-206 that binds to the 3′UTR of FSTL1 mRNA [32].

During mycobacterial infection, miR-32-5p negatively regulates the inflammatory response [119] and promotes the survival of infected macrophages. MiR-32-5p binds to the 3′UTR of FSTL1 decreasing mRNA and protein levels (Fig. 3). Re-expression of FSTL1 completely reverses the inhibitory effects of miR-32-5p on secretion of inflammatory cytokines, indicating that inhibition of FSTL1 is a mediator of the anti-inflammatory effects [33]. However, the increased level of Fslt1 protein seems to have no effect on the level of released cytokines.

Significant lower levels of miR-27a and higher levels of FSTL1 are found in serum, synovial tissue, and fibroblast-like synoviocytes of RA patients compared to healthy controls. Transfection of fibroblast-like synoviocytes with miR-27a inhibits cell migration and invasion and downregulates TLR4, NF-κB, and MMPs. Further analysis showed that these effects are the result of miR-27a-mediated downregulation of FSTL1 expression via its target sequence in the 3′UTR (Fig. 3). Moreover, overexpression of FSTL1 via adenoviral vector rescues the miR-27a effects [24].

## Concluding remarks

From a clinical point of view, it is important to consider that during pathological conditions, such as cancer and cardiovascular disease, not only individual cell behaviors such as proliferation and migration are important but also paracrine communication between cells, inflammation, and vascularization. Because FSTL1 has been shown to be involved in multiple signaling pathways and processes, results observed in specific cell types and in defined conditions should be regarded on their specific merits and not generalized, as yet.

FSTL1 is a secreted glycoprotein of which the expression shifts during pathological conditions. It participates in the regulation of important signaling pathways such as the TGF-β/BMP balance, immune response and Wnt signaling. In humans, its function is tightly regulated. In primates, an additional level of post-transcriptional regulation has been identified; the primary transcript serves as a precursor for the FSTL1 protein or as a pre-microRNA. These alternative functions appear mutually exclusive. Moreover, multiple microRNA-binding sites have been identified in the 3′UTR of FSTL1 mRNA and their role in inhibition of FSTL1 expression has been shown in several models. Finally, at the post-translational level, FSTL1 is also regulated by glycosylation and the structure of the oligosaccharide chain shows species- and cell-specificity-affecting function. It is very important to take all these variables into account during experimental design and in the interpretation of apparently contradictory data reported in literature. Therefore, a future in-depth analysis of the relationship between mRNA expression and functional secreted protein and the composition of the oligosaccharide chain in relation with the observed biological effects of FSTL1 is needed and essential to forward the use of FSTL1 in clinics.

### Electronic supplementary material

Below is the link to the electronic supplementary material.

Supplementary material 1 (DOCX 32 kb)
