# Supplementary material for: Follistatin-like 1 in development and human diseases
Source: Cell Mol Life Sci. 2018 Mar 29;75(13):2339–54. doi: 10.1007/s00018-018-2805-0 (PMC5986856; doi:10.1007/s00018-018-2805-0)
Supplement: Supplementary file 1 — Supplementary material 1 (DOCX 32 kb) [file 18_2018_2805_MOESM1_ESM.docx]

Supplemental Table 1. **Potential MicroRNA binding sites in FSTL1 gene**

List, position and main biological processes of the predicted microRNA binding sites in the 3’UTR of FSTL1 gene. Two different sources has been used: for prediction: [www.microrna.org](http://www.microrna.org) and [www.targetscan.org](http://www.targetscan.org).

For information about the miRNA, [www.miRBase.org](http://www.miRBase.org) was consulted[120]

| **Human microRNA** | **Binding position in 3’UTR** | **Biological relevance** |
| --- | --- | --- |
| miR-1 | 2099; 2380 | CVD |
| miR-15 | 2203 | Leukemia; Adipogenesis |
| miR-16 | 2203 | Leukemia |
| miR-124 | 1908 | Neuronal differentiation |
| miR-1271 | 1755 |  |
| miR-136 | 1521 |  |
| miR-137 | 143 | Neural cell proliferation and differentiation |
| miR-154 | 1165 |  |
| miR-181a/b/c/d | 2466 | Tumourigenesis; Adipogenesis |
| miR-195 | 2203 | Leukemia |
| miR-200 | 83; 1014 |  |
| miR-203 | 1431; 1823 | Epidermal boundary; Psoriasis; Tumourigenesis |
| miR-205 | 140 | Tumourigenesis |
| miR-22 | 2150 | Adipogenesis |
| miR-25 | 142 |  |
| miR-299-3p | 2385 |  |
| miR-29a/b/c | 213; 629 |  |
| miR-300 | 571; 2554 |  |
| miR-328 | 165 |  |
| miR-363 | 142 | Adipogenesis |
| miR-367 | 142 |  |
| miR-381 | 571 |  |
| miR-383 | 1204 |  |
| miR-424 | 2203 |  |
| miR-4262 | 2481 |  |
| miR-429 | 83; 1013 |  |
| miR-485-5p | 411 |  |
| miR-497 | 2203 |  |
| miR-503 | 2203 |  |
| miR-506 | 1907 |  |
| miR-539 | 634 |  |
| miR-544 | 1678 |  |
| miR-613 | 2099; 2382 |  |
| miR-653 | 1504 |  |
| miR-6838 | 2203 |  |
| miR-7 | 2211 | Maintenance of stability under environmental flux |
| miR-873 | 1233 |  |
| miR-876-5p | 2089 |  |
| miR-9 | 623; 1643 | Neuronal differentiation |
| miR-92 | 142 | Tumourigenesis |
| miR-96 | 1753 | Hearing loss |
